# Activation of Signal Transduction and Activator of Transcription 3 Signaling Contributes to *Helicobacter*-Associated Gastric Epithelial Proliferation and Inflammation

**DOI:** 10.1155/2018/9050715

**Published:** 2018-04-08

**Authors:** Yasuaki Ishii, Wataru Shibata, Makoto Sugimori, Yoshihiro Kaneta, Masatomo Kanno, Takeshi Sato, Soichiro Sue, Eri Kameta, Hiroaki Kaneko, Kuniyasu Irie, Tomohiko Sasaki, Masaaki Kondo, Shin Maeda

**Affiliations:** ^1^Department of Gastroenterology, Yokohama City University Graduate School of Medicine, 3-9 Fukuura, Kanazawa-ku, Yokohama 236-0004, Japan; ^2^Advanced Medical Research Center, Yokohama City University, 3-9 Fukuura, Kanazawa-ku, Yokohama 236-0004, Japan

## Abstract

**Background/Aim:**

Although IL-6-mediated activation of the signal transduction and activator of transcription 3 (STAT3) axis is involved in inflammation and cancer, the role of STAT3 in *Helicobacter*-associated gastric inflammation and carcinogenesis is unclear. This study investigated the role of STAT3 in gastric inflammation and carcinogenesis and examined the molecular mechanism of *Helicobacter*-induced gastric phenotypes.

**Methods:**

To evaluate the contribution of STAT3 to gastric inflammation and carcinogenesis, we used wild-type (WT) and gastric epithelial conditional *Stat3*-knockout (*Stat3^Δgec^*) mice. Mice were infected with *Helicobacter felis* and euthanized at 18 months postinfection. Mouse gastric organoids were treated with recombinant IL-6 (rIL-6) or rIL-11 and a JAK inhibitor (JAKi) to assess the role of IL-6/STAT3 signaling *in vitro*.

**Results:**

Inflammation and mucous metaplasia were more severe in WT mice than in *Stat3^Δgec^* mice. The epithelial cell proliferation rate and STAT3 activation were increased in WT mice. Application of rIL-6 and rIL-11 induced expression of intestinal metaplasia-associated genes, such as *Tff2*; this induction was suppressed by JAKi administration.

**Conclusions:**

Loss of STAT3 signaling in the gastric mucosa leads to decreased epithelial cell proliferation, atrophy, and metaplasia in the setting of *Helicobacter* infection. Therefore, activation of STAT3 signaling may play a key role in *Helicobacter*-associated gastric carcinogenesis.

## 1. Introduction

*Helicobacter pylori* infection, a major risk factor for gastric cancer, drives the initiation and progression of mucosal atrophy, intestinal metaplasia, and dysplasia toward gastric cancer via intracellular signaling pathways, such as the interleukin-6- (IL-6-) IL-11-JAK/STAT pathway [1–3]. The IL-6 family of cytokines binds to the *α*-subunit of their specific receptors, associates with gp130 homodimers at the cell membrane, and activates the SHP-2/ERK and JAK/STAT signaling pathways [4–6]. STAT3 signaling regulates various biological processes, such as cell growth, survival, differentiation, and apoptosis [7]. STAT3 may act as an oncogene, as it is aberrantly activated in various human malignancies [8]. Furthermore, an epigenetic mechanism of crosstalk between STAT3 and nuclear factor kappaB (NF-*κ*B) is associated with STAT3 activation in malignancy, suggesting a role for inflammation in carcinogenesis [9–11]. NF-*κ*B/IL-6/STAT3 plays an important role in inflammatory carcinogenesis [10]. We previously reported that NF-*κ*B/IL-6 induces liver metastasis [12] and gastric cancer [13].

Inflammation can initiate carcinogenesis in various organs, and continuous activation of STAT3 plays an important role in the initiation of inflammation and cellular transformation in gastric cancer and in several other cancers [8, 14]. For example, the IL-6 family of proinflammatory cytokines and their downstream effector STAT3 are important regulators in colitis-associated colon cancer [15, 16], and the STAT3 signaling pathway contributes to inflammation-associated gastric carcinogenesis [17, 18].

In the gastric mucosa, IL-6 is upregulated upon *H. pylori* infection and contributes to gastric tumorigenesis [13, 19, 20]. Although activation of STAT3 induced by *Helicobacter* has been reported in gastric cancer cell lines and *H. felis*-infected mice [21], the precise role of STAT3 in *Helicobacter*-induced gastric inflammation and metaplasia is unclear. Because gastric carcinogenesis results from prolonged gastritis due to long-term *Helicobacter* infection, we investigated the role of STAT3 in gastric carcinogenesis using *Stat3^Δgec^* mice with long-term *Helicobacter* infection [22–24]. We also used a gastric organoid culture system to assess the mechanism(s) underlying inflammation-associated metaplasia and cancer.

## 2. Methods

### 2.1. Mice

All animals were maintained at Yokohama City University Graduate School of Medicine. *Foxa3-cre* mice were a gift from Professor Klaus H. Kaestner and were used to direct expression of *cre* recombinase to the gastric mucosa [25]. *Stat3^f/f^* mice were purchased from Oriental BioService Inc. (Kyoto, Japan). *Stat3^Δgec^* mice were established by crossing *Foxa3-cre* mice with *Stat3^f/f^* mice. We used *cre*-negative *Stat3^f/f^* mice as a WT control.

### 2.2. Bacterial Culture

*H. felis* ATCC 49179 has been described previously [26]. In brief, *H. felis* was cultured for 48 h at 37°C under microaerobic conditions on 5% sheep blood agar supplemented with antibiotics. Bacteria were aliquoted at 10^10^ colony-forming units/mL in trypticase soy broth with 10% glycerol and stored at −70°C.

### 2.3. Chronic *H. felis* Infection Model

WT and *Stat3^Δgec^* mice were inoculated with *H. felis* or with sterile broth as a control. Inocula (0.2 mL, 10^10^ colony-forming units/mL) were delivered by oral gavage three times per week using a sterile gavage needle. Mice were euthanized at 18 months postinfection. At necropsy, stomachs were removed *en bloc*, opened, and emptied. Mice were examined for gross changes. Stomach tissue specimens were fixed in neutral-buffered 10% formalin, processed by standard methods, embedded in paraffin, sectioned at 4 *μ*m, and stained with hematoxylin and eosin (H&E). Infection status was confirmed pathologically. Additional sections were cut for immunohistochemistry, and additional samples were processed for PCR analysis.

### 2.4. Histological Evaluation

Two researchers who are familiar with mouse stomach pathology (YI and WS) were blind to the mouse genotype and independently scored histopathological changes on an ordinal scale from 0 to 4 with a previously reported histopathologic grading scheme. It is recapitulation of the primary histopathologic features that define the human disease in *Helicobacter*-associated gastritis and neoplasia for a mouse model [27]. In brief, inflammation, mucous metaplasia, oxyntic gland atrophy, and pseudopyloric metaplasia in the gastric corpus were scored as increasing and extension of leukocyte, foci replacing of parietal cells, loss of chief/parietal cells, and site of foci replacing.

### 2.5. Immunohistochemical Examination

After deparaffinization and rehydration, endogenous peroxidase was blocked with 3% hydrogen peroxide for 20 min at room temperature. For heat-mediated antigen retrieval, slides were processed for 15 min at 121°C in an autoclave in a 10 mM citrate buffer (pH 6.0). For immunohistochemistry using the anti-phospho(p)-tyrosin(Y)-STAT3 antibody, samples were processed in 1 mM ethylenediaminetetraacetic acid (EDTA) for 20 min. Slides were incubated with primary antibodies according to the manufacturer's directions at 4°C overnight. The following primary antibodies were used: anti-STAT3 (124H6) (mouse monoclonal, 1 : 600, Cell Signaling Technology, Danvers MA, USA), anti-p-Y-STAT3 (D3A7) (rabbit monoclonal, 1 : 400, Cell Signaling Technology), anti-Ki67 (SP6) (rabbit monoclonal, 1 : 100, Abcam, Cambridge, MA, USA), trefoil factor 2 (TFF2) (polyclonal, 1 : 200, Proteintech, Rosemont, IL, USA), and anti-CD44v6 (9A4) (rat monoclonal, 1 : 100, Bio-Rad Company, Berkeley, CA, USA). Secondary anti-rabbit, anti-rat, and anti-mouse antibodies (Vector Laboratories, Burlingame, CA, USA) were diluted 1 : 200 and applied to the samples for 30 min at room temperature. The solutions in the VECTASTAIN ABC kit (Vector Laboratories) were diluted 1 : 200 according to the manufacturer's directions.

### 2.6. Gastric Organoid Culture

We followed the culture methods according to previously described [28]. In brief, uninfected WT mice and *Stat3^Δgec^* mice were euthanized. The antrum was removed and shaken at 4°C for 3 h in 0.1 M EDTA. Gastric epithelial cells were dissected, washed with phosphate-buffered saline (PBS; Life Technologies Inc.), and centrifuged, and the pellets were resuspended with IntestiCult (STEMCELL Technologies Inc., Vancouver, Canada). Resuspended pellets were transferred to 24-well plates (Sumitomo Bakelite Co., Tokyo, Japan) coated with 2% Matrigel (Corning, NY, USA) and stored at 37°C in a 5% CO_2_ incubator (Supplement Figure 2).

### 2.7. Stimulation of Gastric Organoids with IL-6 or IL-11 and JAKi

Four days after removal of gastric organoids from WT mice and *Stat3^Δgec^* mice, cells were treated with 1 *μ*M JAKi or culture medium as a control. After incubation for 1 h, the organoids were washed and treated with 40 ng/mL rIL-6, rIL-11, or PBS and incubated for 4 h. Next, RNA was extracted using an RNeasy mini kit (Qiagen, Limburg, The Netherlands). Recombinant mouse rmIL-6 and rmIL-11 were purchased from PeproTech, and JAK inhibitor (JAKi) was obtained from Calbiochem (Darmstadt, Germany) [29].

### 2.8. Quantitative Real-Time Polymerase Chain Reaction

RNA was extracted from the mouse antrum using ISOGEN2 (Nippon Gene, Tokyo, Japan) following the manufacturer's directions. RNA was extracted from gastric organoids using an RNeasy mini kit. cDNA was generated using a high-capacity RNA-to-cDNA kit (Thermo Fisher Scientific, Waltham, MA, USA). Quantitative real-time polymerase chain reaction (qRT-PCR) amplification of cDNA was performed in duplicate using Fast SYBR Green Master Mix (Thermo Fisher Scientific) and a 7900HT Fast Real-Time PCR System (Applied Biosystems, Waltham, MA, USA). qRT-PCR was performed using the following conditions: 95°C for 15 min, followed by 45 cycles of 95°C for 15 s, 55°C for 30 s, and 72°C for 30 s. The following primers were used: *cyclinD1* (F: gctgcaaatggaactgcttctggt, R: taccatggagggtgggttggaaat), CDX2 (F: gctgccacacttgggctctc, R: cggctgaggctgggaaggtt), *Tff2* (F: gcagtgctttgatcttggatgc, R: tcaggttggaaaagcagcagtt), *Itln1* (F: tgctaccagaggttgcagtg, R: tgctcctgcttgatttcctt), *ATP6v0d2* (F: ggaagctgtcaacattgcaga, R: tcaccgtgatccttgcagaat), and *Gapdh* (F: gacatcaagaaggtggtgaagcag, R: ataccaggaaatgagcttgacaaa).

### 2.9. Immunoblotting

Proteins were separated by sodium dodecyl sulfate-polyacrylamide gel electrophoresis (SDS-PAGE) (e-PAGEL, ATTO, Tokyo, Japan), transferred to nitrocellulose membranes, and incubated with the following primary antibodies: anti-STAT3 (1 : 1000, rabbit; Cell Signaling Technology), anti-p-Y-STAT3 (1 : 1000, rabbit; Cell Signaling Technology), anti-GAPDH (1 : 2000, rabbit; Cell Signaling Technology), and anti-CDX2 (1 : 1000, rabbit; Abcam). The blots were next incubated with the appropriate secondary antibodies, and proteins were detected using the ECL Prime Western blotting detection reagent (GE Healthcare, Buckinghamshire, UK). Images were captured using an LAS-3000 imaging system (Fujifilm, Tokyo, Japan).

### 2.10. Confirmation of *Foxa3-cre* Recombination of STAT3 Locus

PCR analysis was carried out using genomic DNA extracted from the organoids prepared from epithelial gastric cell as described above using ReliaPrep gDNA tissue miniprep system (Promega Corporation, Fitchbrug, WI, USA) in order to confirm whether recombination was specifically achieved in gastric epithelial cells.

PCR was performed using the following conditions: 95°C for 10 min, followed by 35 cycles of 95°C for 30 s, 55°C for 30 s, and 72°C for 30 s. The following primers were used: *Stat3* (a: cctgaagaccaagttcatctgtgtgac, b: cacacaagccatcaaactctggtctcc, and c: gatttgagtcagggatccataacttcg).

### 2.11. Statistical Analysis

Results are expressed as means ± standard error unless otherwise stated. Student's *t*-test was used to evaluate statistical significance. Values of *p* < 0.05 were considered to indicate statistical significance.

## 3. Results

### 3.1. Generation of *Stat3^Δgec^* Mice and *H. felis* Infection

Unlike knockout mice of other STAT proteins, *Stat3*-deficient mice die during early embryogenesis [30]. Therefore, to identify the mechanism by which *Stat3* affects gastric epithelial inflammation and carcinogenesis, we generated WT and *Stat3^Δgec^* mice by crossing *Foxa3-cre* mice with *Stat3^f/f^* mice. Recombination was confirmed using genomic DNA from gastric organoid which is made of gastric epithelial cells (Supplement Figure 1).

**Figure 1:**
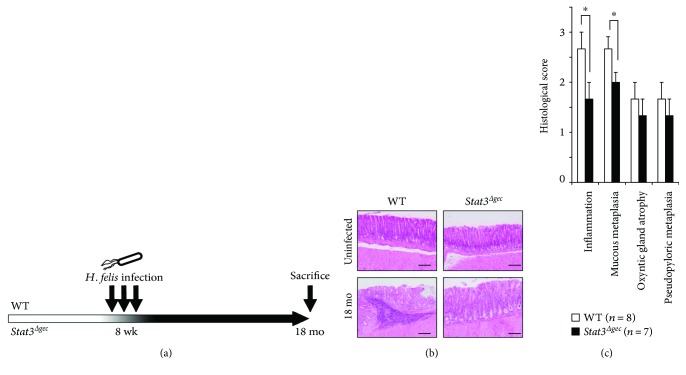
Mouse model of infection and H&E staining of the mouse gastric mucosa. (a) Eight-week-old mice were infected with *H. feli*s three times every other day and were euthanized at 18 months postinfection. (b) Uninfected control mice with WT and *Stat3^Δgec^* mice were sacrificed at 18 months (*n* = 6 each). WT and *Stat3^Δgec^* mice infected with *H. felis* for 18 months (*n* = 8 WT and *n* = 7* Stat3^Δgec^*). Representative H&E-stained images are shown (magnification ×100, scale bar 100 *μ*m). (c) Histological scores at 18 months postinfection. Each parameter was scored on an ordinal scale from 0 to 4 (^∗^ *p* < 0.05).

*Stat3^Δgec^* mice were healthy, and no evidence of growth disturbance was detected during the observation period in the absence of *H. felis* infection (data not shown). Mice were infected with *H. felis*, which is associated with gastric carcinogenesis, and were euthanized at 18 months postinfection. Uninfected mice were euthanized at the same age as the controls (Figure 1(a)).

### 3.2. Histological Changes

H&E staining of uninfected WT and *Stat3^Δgec^* mice did not show gastric inflammation (Figure 1(b), top). In the presence of *Helicobacter* infection, all mice showed gastric inflammation with lymph follicles, neck cell hyperplasia, oxyntic atrophy, and mucous metaplasia at 18 months postinfection (Figure 1(b), bottom). *Helicobacter* colonization was similar in WT and *Stat3^Δgec^* mice (data not shown). No mice developed cancer at 18 months postinfection.

The histological inflammation score (WT versus *Stat3^Δgec^*; 2.7 versus 1.7, *p* < 0.05) and mucous metaplasia score (WT versus *Stat3^Δgec^*; 2.7 versus 1.9, *p* < 0.05) were lower in *Stat3^Δgec^* than in WT mice; however, other parameters, such as oxyntic gland atrophy and pseudopyloric metaplasia, were not significantly different (Figure 1(c)).

### 3.3. Phosphorylation of STAT3 in *H. felis*-Infected Mice

**Figure 2:**
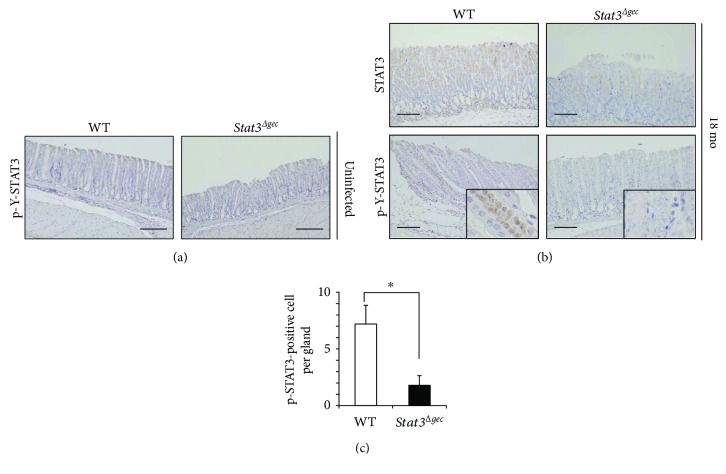
Immunohistochemistry of WT and *Stat3^Δgec^* mice. (a) Immunohistochemistry for phospho-STAT3 in uninfected control mice (magnification ×200, scale bar 50 *μ*m). (b) Immunohistochemistry for STAT3 and phospho-STAT3 in mice infected with *H. felis* at 18 months (magnification ×200 (inset ×400), scale bar 50 *μ*m). (c) Number of p-Y-STAT3-positive cells per gland in *Stat3^Δgec^* and WT mice (*n* = 30 glands each) at 18 months postinfection (^∗^ *p* < 0.05).

Immunohistochemistry was performed to assess the expression and activation of STAT3 and other markers associated with *Helicobacter* gastritis. STAT3 phosphorylation was not detected in uninfected mice at 18 months postinfection (Figure 2(a)). In contrast, STAT3 phosphorylation was detected in gastric epithelial cells at 18 months postinfection and was significantly more pronounced in WT mice than in *Stat3^Δgec^* mice (Figures 2(b) and 2(c)).

### 3.4. Cell Proliferation in *H. felis*-Infected Mice

**Figure 3:**
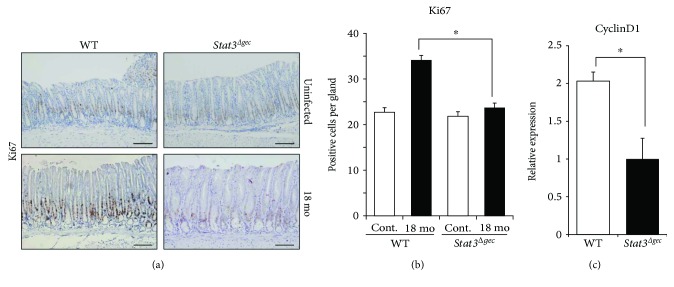
Cell proliferation rate in *Stat3^Δgec^* mice. (a) Immunohistochemistry for Ki67 in WT and *Stat3^Δgec^* mice. Uninfected (magnification ×200, scale bar 50 *μ*m) (top) and infected mice at 18 months (magnification ×200, scale bar 50 *μ*m) (bottom). (b) Proliferation of gastric epithelial cells as determined by Ki67 staining at 18 months postinfection with *H. felis* (^∗^ *p* < 0.05). Uninfected mice were used as controls (*n* = 30 glands each). (c) CyclinD1 mRNA levels in the stomachs of *Stat3^Δgec^* and WT mice at 18 months postinfection with *H. felis* (^∗^ *p* < 0.05).

Ki67 staining and quantification of cyclinD1 mRNA levels of gastric tissue were performed to assess cell proliferation. The proliferation rate of gastric epithelial cells was lower in *Stat3^Δgec^* mice than in WT mice at 18 months postinfection (Ki67-positive cells per gland; WT versus *Stat3^Δgec^*: 34.1 versus 23.7, *p* < 0.05) (Figures 3(a) and 3(b)). The cyclinD1 mRNA level was markedly lower in *Stat3^Δgec^* mice compared to WT mice (Figure 3(c)). These results suggest that STAT3 is involved in the proliferation of gastric epithelial cells.

### 3.5. Spasmolytic Polypeptide-Expressing Metaplasia Was Suppressed in *Stat3^Δgec^* Mice

**Figure 4:**
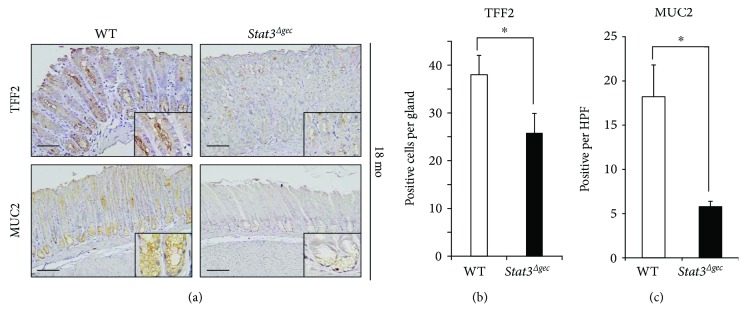
TFF2 and MUC2 protein levels in the mouse stomach. (a) Immunohistochemistry for TFF2 (top) and MUC2 (bottom) in WT and *Stat3^Δgec^* mice infected with *H. felis* for 18 months (magnification ×200 (inset ×400), scale bar 50 *μ*m). (b) Number of TFF2-positive cells per gland in *Stat3^Δgec^* and WT mice (*n* = 30 glands each) at 18 months postinfection (^∗^ *p* < 0.05). (c) Number of MUC2-positive cells per high-power field in *Stat3^Δgec^* and WT mice (*n* = 30 glands each) at 18 months postinfection (^∗^ *p* < 0.05).

To assess the contribution of STAT3 to intestinal metaplasia (IM), we analyzed the expression of trefoil factor 2 (TFF2), a marker of spasmolytic polypeptide-expressing metaplasia (SPEM), using immunohistochemistry [31]. At 18 months postinfection, the TFF2 protein level was significantly higher in WT mice compared to *Stat3^Δgec^* mice (TFF2-positive cells per gland; WT versus *Stat3^Δgec^* = 38.0 ± 4.05 versus 25.8 ± 3.87) (Figures 4(a) and 4(b)). The protein level of MUC2, a marker of IM, was also significantly higher in WT mice compared to *Stat3^Δgec^* mice (MUC2-positive cells per high-power field; WT versus *Stat3^Δgec^* = 18.3 versus 5.3) (Figures 4(a) and 4(c)). Therefore, STAT3 activation may play an important role in the development of SPEM/IM due to *H. felis* infection.

### 3.6. Role of JAK/STAT Signaling in the Development of Metaplasia

We next investigated the role of JAK/STAT signaling in the development of metaplasia using a gastric organoid culture system. We compared the organoids of WT mice with the organoids of *Stat3^Δgec^* mice. Each organoid was stimulated with rmIL-6 or rmIL-11.

**Figure 5:**
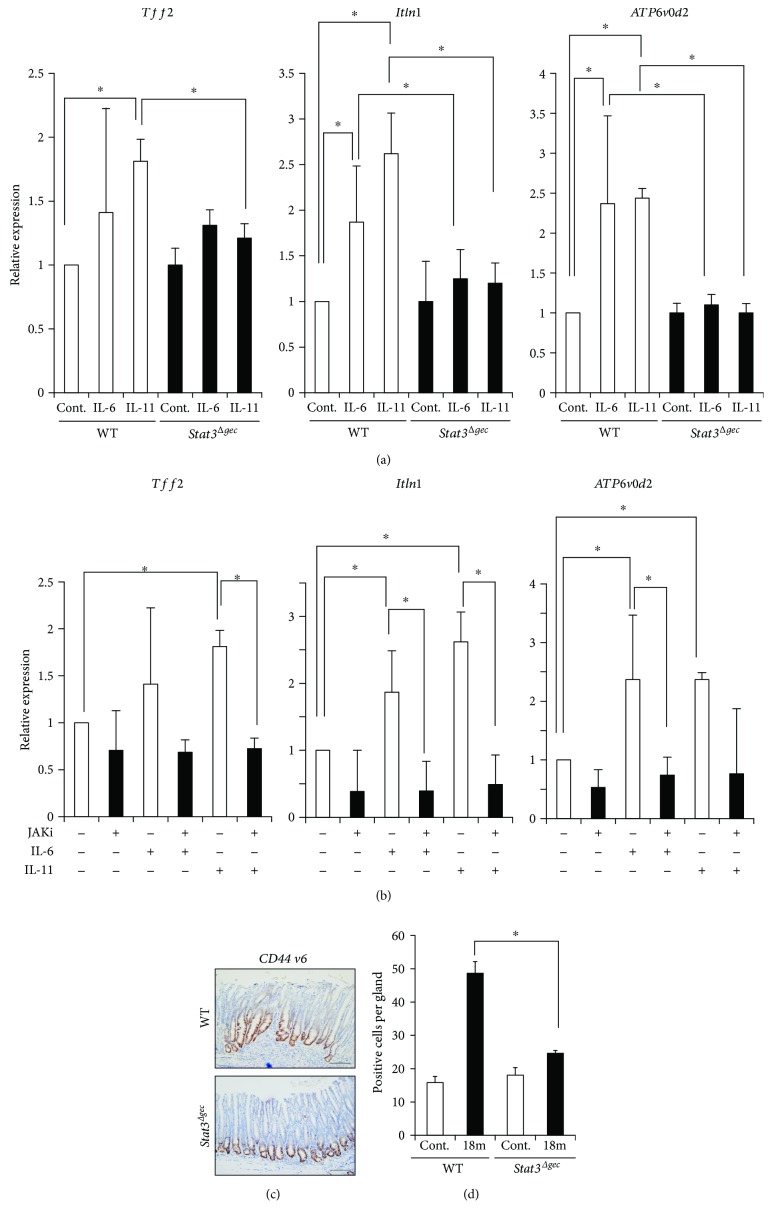
Contribution of STAT3 signaling to the proliferation in gastric stem/progenitor cells. (a) Expression of intestinal metaplasia-associated genes in gastric organoids of *Stat3^Δgec^* or WT mice treated after stimulation with recombinant IL-6 or IL-11 (^∗^ *p* < 0.05) (*n* = 6 organoids of WT mice, *n* = 3 organoids of *Stat3^Δgec^* mice). (b) Expression of intestinal metaplasia-associated genes in gastric organoids treated with or without a JAK inhibitor after stimulation with recombinant IL-6 or IL-11 (^∗^ *p* < 0.05) (*n* = 6 each). (c) Immunohistochemistry for CD44v6 in WT and *Stat3^Δgec^* mice infected with *H. felis* for 18 months (magnification ×200, scale bar 50 *μ*m). (d) Number of CD44v6-positive cells per gland in *Stat3^Δgec^* and WT mice at 18 months postinfection (^∗^ *p* < 0.05). Uninfected mice were used as controls (*n* = 30 glands each).

The expression of *Tff2*, a SPEM marker, was significantly increased by stimulation with rmIL-11 and was suppressed in the organoids of *Stat3^Δgec^* mice by JAKi, an inhibitor of STAT3 signaling. In contrast, stimulation with rmIL-6 did not increase *Tff2* expression. We also assessed the involvement of *Stat3* signaling in the expression of the IM-associated gene, *Intelectin1* (*Itln1*), and lysosomal H^+^ transporting ATPase subunit (*ATP6v0d2*), which is expressed in intestinal goblet cells and intestinal metaplasia but not in normal gastric tissue [32]. *Itln1* and *ATP6v0d2* expression was increased by IL-6 or IL-11 and downregulated by inhibition of JAK/STAT signaling (Figure 5(b)).

Finally, we assessed expression of the putative gastric stem/progenitor marker CD44 in the mouse stomach using immunohistochemistry. CD44 expression was significantly more pronounced in WT mice compared to *Stat3^Δgec^* mice (CD44-positive cells per gland; WT versus *Stat3^Δgec^* = 48.6 ± 3.5 versus 24.6 ± 0.8) (Figures 5(c) and 5(d)). Therefore, STAT3 signaling contributes to the proliferation of gastric stem/progenitor cells *in vivo*.

## 4. Discussion

In this study, we established mice with conditional *Stat3* knockout in gastric epithelial cells and evaluated the role of STAT3 in *Helicobacter*-induced gastric inflammation and metaplasia. Both histological changes and STAT3 phosphorylation were less marked in *Stat3^Δgec^* mice compared to WT mice.

Loss of *Stat3* in gastric epithelial cells reduces proliferation and SPEM, suggesting that STAT3 induces *Helicobacter*-associated gastric inflammation and IM. C57BL/6 mice are unstable for colonization by *H. pylori* [33], infection by which leads to gastric SPEM, dysplasia, and invasive cancer [34]. Therefore, we infected mice with *H. felis* instead of *H. pylori* [35]. *TFF2* expression was downregulated in *Stat3^Δgec^* mice, suggesting that STAT3 regulates SPEM; this is in agreement with previous reports [36].

STAT3 reportedly acts as an oncogene [8]. To assess the role of STAT3 in gastric carcinogenesis, we induced gastric cancer in *Stat3^Δgec^* mice using the chemical carcinogen *N*-methyl-*N*-nitrosourea (MNU) [33, 37, 38]. WT and *Stat3^Δgec^* mice were administered MNU and sacrificed 40 weeks later. Dysplasia and/or carcinoma developed in the antrum of WT and *Stat3^Δgec^* mice. Although there was no obvious difference in tumor size, WT mice exhibited a larger number of tumors than *Stat3^Δgec^* mice; however, this difference did not reach statistical significance (under submission). Therefore, gastric epithelial STAT3 may contribute to cancer initiation; however, JAK/STAT signaling in gastric epithelial cells, *Stat3* expression in stromal cells, and/or other signaling pathways (e.g., the JNK and NF-*κ*B signaling pathways) may contribute to gastric tumorigenesis [38, 39].

Treatment with JAKi decreased the expression of IM-associated genes. Trastuzumab, a monoclonal antibody that acts on the HER2/neu (erbB2) receptor, is currently used to treat HER2-positive advanced gastric cancer [40]. Inhibition of STAT3 has also been used as a novel treatment option for gastric cancer and cancers in other organs [41]. Boston Biomedical Inc. conducted a phase 3 clinical trial of the STAT3 inhibitor BBI608 in patients with advanced, previously treated gastric, and gastroesophageal junction adenocarcinoma. However, this trial failed, which was, in part, due to the failure to consider p-Y-STAT3 expression in patient recruitment. Thus, STAT3 inhibitors may be effective only in patients with p-STAT3-positive gastric cancer. Indeed, analysis of a phase 3 clinical trial of BBI608 in patients with colorectal cancer was announced by Dr. D. J. Jonker at the “European Society for Medical Oncology 2016” and showed that STAT3 phosphorylation-positive patients exhibited significantly improved survival.

In this study, we investigated the activation of STAT3 using *Stat3^Δgec^* mice; however, other factors are involved in *Helicobacter*-induced gastritis/carcinogenesis, for example, stromal cells have been prominently increased in infected gastritis [23, 42]. During the early stages of inflammation, *α*SMA-positive myofibroblasts invade the gastric mucosa, where they induce proliferation of epithelial cells. Additionally, the proinflammatory chemokine SDF-1 is associated with the remodeling of stem cell niches in the bone marrow [23]. Conditional knockout mice in macrophages and neutrophils exhibit increased production of proinflammatory cytokines [43]. We reported previously that proinflammatory cytokines and chemokines exert conflicting effects on epithelial cells and stromal cells in hepatocellular carcinoma [44]. Therefore, the effects of STAT3 loss in inflammatory cells should be evaluated using *cre* mice.

The role of cytokine signaling between stem/progenitor cells and their niche has recently been a focus of research [45]. Therefore, we evaluated stem cell behavior by treating gastric organoids with JAKi. Cytokine stimulation increased the expression of factors associated with SPEM, IM, and gastric cancer; this increased expression was suppressed by administration of JAKi. CDX1/2 reportedly converts gastric epithelial cells into tissue stem/progenitor cells, which then transdifferentiate into intestinal epithelial cells [46]. Therefore, STAT3 may affect the expansion of gastric stem/progenitor cells by inducing expression of Cdx2. Indeed, there were more CD44-positive cells in WT than *Stat3^Δgec^* mice [47, 48].

In summary, activation of *Stat3* induces inflammation and IM in the setting of *Helicobacter* infection and cytokine stimulation triggers STAT3 activation and IM *in vitro*. Therefore, our results suggest that activation of STAT3 signaling plays a key role in *Helicobacter*-associated gastric inflammation and IM.

## Supplementary Materials

Supplementary 1 Supplement Figure 1: PCR analysis of genomic DNA from organoid. A. Map of the WT STAT3 locus, the targeted STAT3 locus of *Stat3^Δgec^* mice.

Supplementary 2 Supplement Figure 2: gastric organoids. A. Gastric organoids from WT mice (top) and *Stat3^Δgec^* mice (bottom). Both grew well and were not recognized as having a deformed shape.
